# Age-sex distribution of patients with high-sensitivity troponin T levels below the 99th percentile

**DOI:** 10.18632/oncotarget.20328

**Published:** 2017-08-18

**Authors:** Jie-Yin Liu, Qiao-Wei Jia, Xiao-Ling Zang, Rong-Hu Wang, Chun-Jian Li, Lian-Sheng Wang, Wen-Zhu Ma, Zhi-Jian Yang, En-Zhi Jia

**Affiliations:** ^1^ First Affiliated Hospital of Nanjing Medical University, Nanjing 210029, Jiangsu Province, China

**Keywords:** high-sensitivity troponin T, the 99th percentile, myoglobin, creatine kinase-MB

## Abstract

**Background:**

Recently, very low concentrations of high-sensitivity cardiac troponin T (hs-cTnT), below the 99th percentile, have been used to immediately exclude acute myocardial infarction in certain patients without taking their age and sex into consideration.

**Results:**

The hs-cTnT values below the 99th percentile (≤ 14 ng/L) were higher in men (*p* = 0.000) and significantly increased with age (*p* = 0.000) among both men and women. In addition, hs-cTnT was positively associated with age (*r* = 0.459, *p* = 0.000), myoglobin (*r* = 0.392, *p* = 0.000), and creatine kinase-MB (*r* = 0.133, *p* = 0.000). Moreover, males were younger (*p* = 0.001) and had higher myoglobin (*p* = 0.000) and creatine kinase-MB (*p* = 0.000) concentrations than females.

**Materials and Methods:**

A total of 5585 consecutive subjects who presented with non-traumatic chest pain/discomfort to the inpatient, outpatient, or emergency department and who underwent high-sensitivity troponin T, myoglobin and creatine kinase-MB testing at presentation, with hs-cTnT below the 99thpercentile (≤ 14 ng/L), were eligible for enrollment.

**Conclusions:**

We suggest that patients’ age, sex and levels of myocardial injury biomarkers should be taken into consideration when ruling out acute myocardial infarction and/or adverse prognostic implications in patients who have very low hs-cTnT concentrations.

## INTRODUCTION

Recently, high-sensitivity cardiac troponin T (hs-cTnT) assays have been implemented worldwide for the diagnosis of acute myocardial infarction (AMI) [1]. In addition to having greater analytical sensitivity, these assays have higher diagnostic sensitivity when used in subjects with suspected myocardial infarction (MI) [2]. According to the third universal definition of MI, the upper reference limit for the hs-cTnT assays is defined as the 99th percentile cut-off value (14 ng/L) in a normal reference population [3, 4]. However, even with high-sensitivity assays, using the conventional cutoff of the 99th percentile results in a diagnostic sensitivity of only approximately 90% at presentation, meaning that serial testing remains necessary before AMI can be excluded [5]. In a large cohort of patients who were admitted to the emergency department (ED) for chest pain, undetectable hs-cTnT levels (≤ 5 ng/L) combined with no signs of ischemia on an electrocardiograph (ECG) indicated a minimal risk of AMI (negative predictive value, NPV: 99.8%) or death within 30 days (negative predictive value, NPV: 100%); Therefore, these patients could be safely discharged directly from the ED [6]. All patients with initial hs-cTnT concentrations that are detectable but below the 99th percentile (≤ 14 ng/L) also have a worse cardiovascular prognosis than those with undetectable troponin (≤ 5 ng/L). Additionally, hs-cTnT concentrations between the limit of blank (LoB) and limit of detection (LoD) (3–4.99 ng/L) are associated with a higher prevalence of traditional risk factors, more cardiac pathology, and worse outcomes than hs-cTnT concentrations below the LOB (3 ng/L), suggesting that cardiac comorbidities can lead to minor increases in hs-cTnT values that may be within the reference range [7, 8].

There have been studies on patients with hs-cTnT values below the 99th percentile, and the clinical implications of such low hs-cTnT concentrations have become increasingly important. However, the cut-off points tested in these studies did not take into account the subjects’ age and sex or include myocardial injury biomarkers such as myoglobin (Myo) and creatine kinase-MB (CK-MB). Therefore, in the present study, we analyzed troponin values measured via hs-cTnT assay in a large, independent hospital-based cohort; estimated the age and sex differences in the cohort with hs-cTnT values below the 99th percentile; and determined the association of low hs-cTnT values with other biochemical markers, such as Myo and CK-MB.

## RESULTS

**Figure 1:**
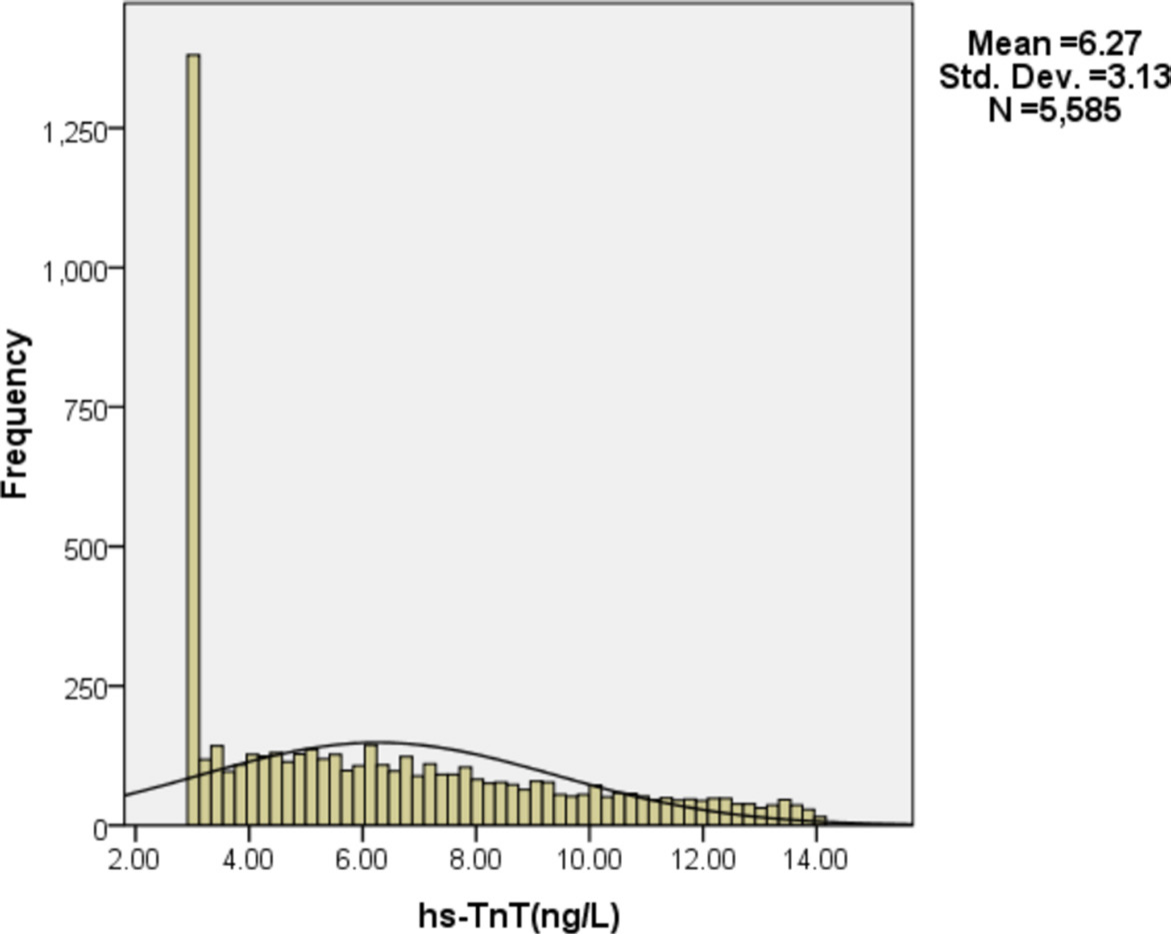
Distribution of hs-cTnT levels below the 99th percentile (14 ng/L)

Figure 1 shows the distribution of hs-cTnT levels below the 99th percentile (14 ng/L) of all the 5585 consecutive subjects. The mean of the hs-cTnT levels was 6.27 ng/L and the standard deviation was 3.13 ng/L.

### Characteristics of the study population stratified by gender

**Figure 2:**
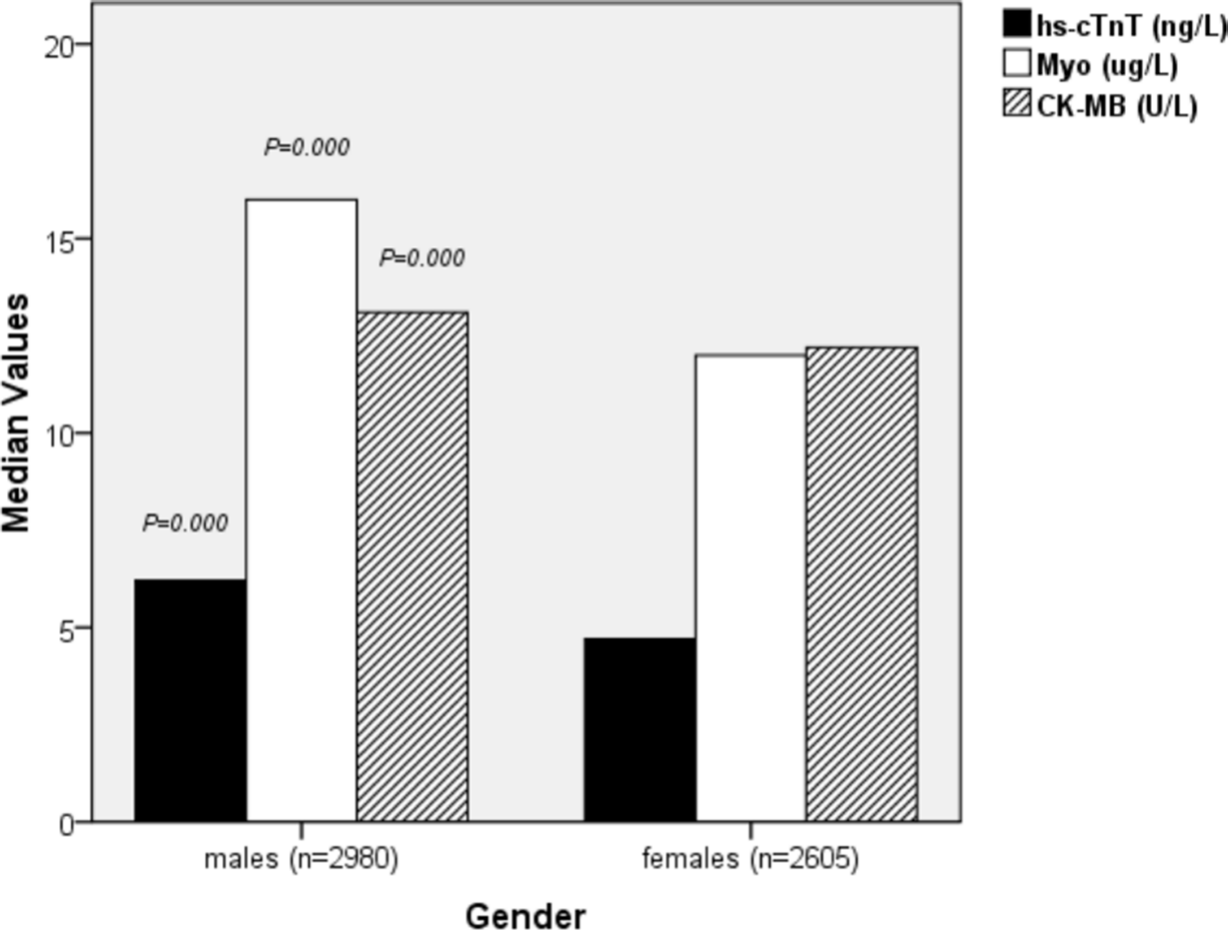
Characteristics of the study population stratified by gender

Table 1 and Figure 2 present the characteristics of the study population. A total of 2980 males and 2605 females were enrolled in the study. As expected, values for hs-cTnT, Myoand CK-MB were significantly different between the two genders. In addition, the values below the 99th percentile upper reference limit (14 ng/L) for hs-cTnT (*p* = 0.000) were higher in men compared with women. Moreover, the male group was younger (*p* = 0.001) and had higher Myo (*p* = 0.000) and CK-MB (*p* = 0.000) concentrations .

**Table 1:** Characteristics of the study population stratified by gender

**Characteristic**	**Males** **(*n =* 2980)**	**Females** **(*n =* 2605)**	**Statistical parameter**	***p* value**
Age (years)	60.00 (48.00–69.00)	61.00 (49.00–71.00)	−3.210	0.001
hs-cTnT (ng/L)	6.21 (3.96–9.05)	4.71 (3.00–7.60)	−14.438	0.000
Myo (ug/L)	16.00 (11.00–25.00)	12.00 (9.00–11.00)	−16.322	0.000
CK-MB (U/L)	13.10 (9.90–16.90)	12.20 (9.10–16.10)	−5.455	0.000

The data are presented as the 50th (25th/75th) percentiles for continuous variables. hs-cTnT, high-sensitivity cardiac troponin T; Myo, myoglobin; and CK-MB, creatine kinase-MB.

### Characteristics of the study population stratified by quartile of age

**Figure 3:**
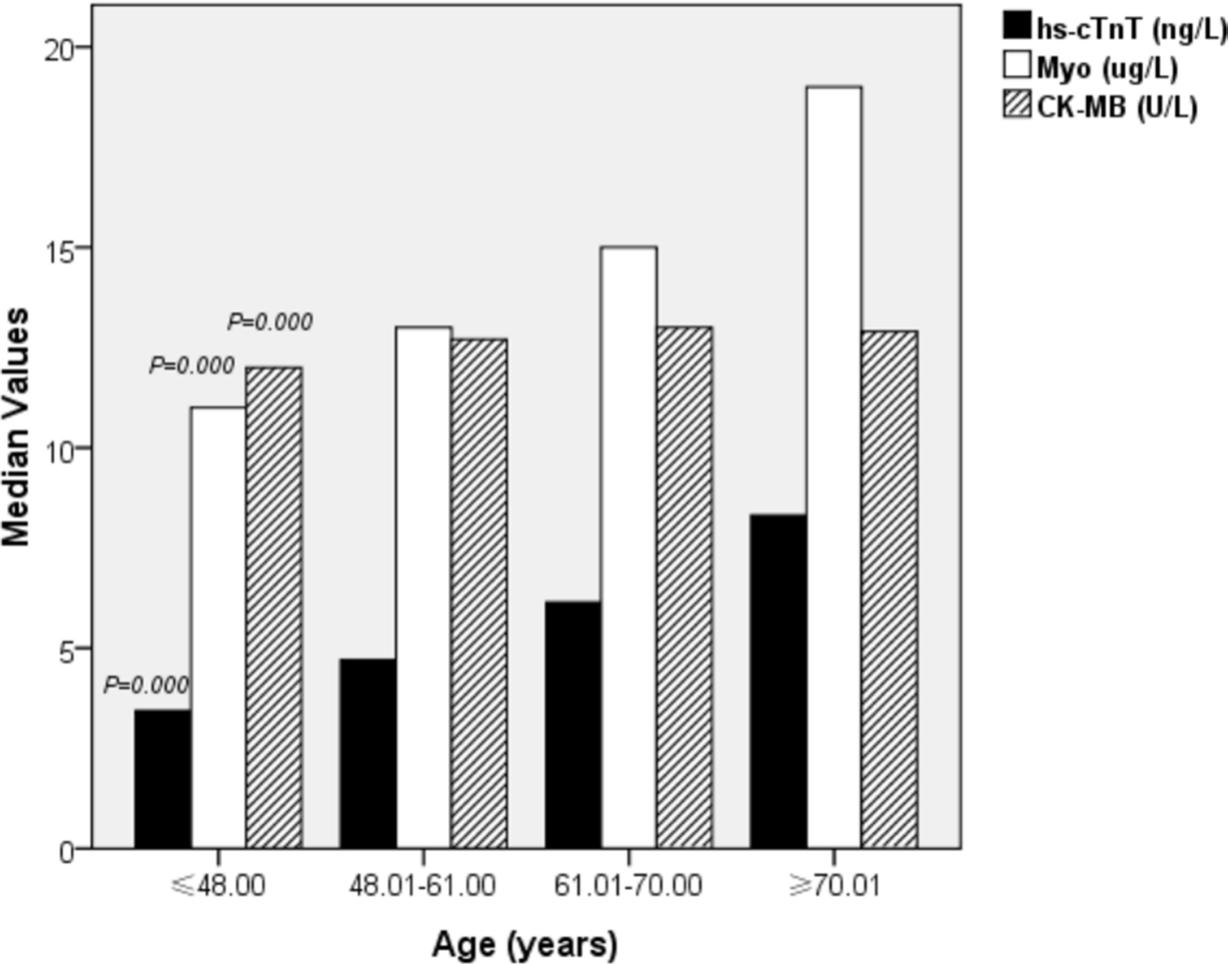
Characteristics of the study population stratified by quartiles of age

Subjects were divided into 4 groups according to the quartile of their age: ≤ 48.00 (first quartile; n=1400 subjects), 48.01–61.00 (second quartile; *n* = 1553 subjects), 61.01–70.00 (third quartile; *n* = 1312 subjects) and ≥ 70.01 (fourth quartile; *n* = 1320 subjects). The characteristics of the study population stratified by quartile of age are shown in Table 2 and Figure 3. Values for hs-cTnT (*p* = 0.000) and Myo (*p* = 0.000) significantly increased with age in both men and women. Age differences were significant among the CK-MB values (*p* = 0.000). In addition, the proportion of males (*p* = 0.006) was significantly greater than that of females in each quartile of age.

**Table 2:** Characteristics of the study population stratified by quartile of age

Characteristics	Age (years)				Statistical parameter	*p* value
	< 48.00 (*n =* 1400)	48.01–61.00 (*n =* 1553)	61.01–70.00 (*n =* 1312)	> 70.01 (*n =* 1320)		
Gender (M/F) hs-cTnT (ng/L)	789/611 3.44 (3.00–5.64)	848/705 4.71 (3.00–7.18)	679/633 6.15 (4.05–8.67)	664/656 8.32 (6.02–10.77)	12.334 1134.813	0.006 0.000
Myo (ug/L)	11.00 (7.00–17.00)	13.00 (10.00–20.00)	15.00 (11.00–22.00)	19.00 (13.00–28.00)	510.210	0.000
CK-MB (U/L)	12.00 (8.80–15.90)	12.70 (9.80–16.60)	13.00 (9.90–17.40)	12.90 (9.53–16.60)	29.673	0.000

The data are presented as the 50th (25th/75th) percentiles for continuous variables and as N1/N2 for binary variables. Hs-cTnT, high-sensitivity cardiac troponin T; Myo, myoglobin; and CK-MB, creatine kinase-MB.

### Spearman correlations of hs-cTnT with age, Myo and CK-MB

**Figure 4:**
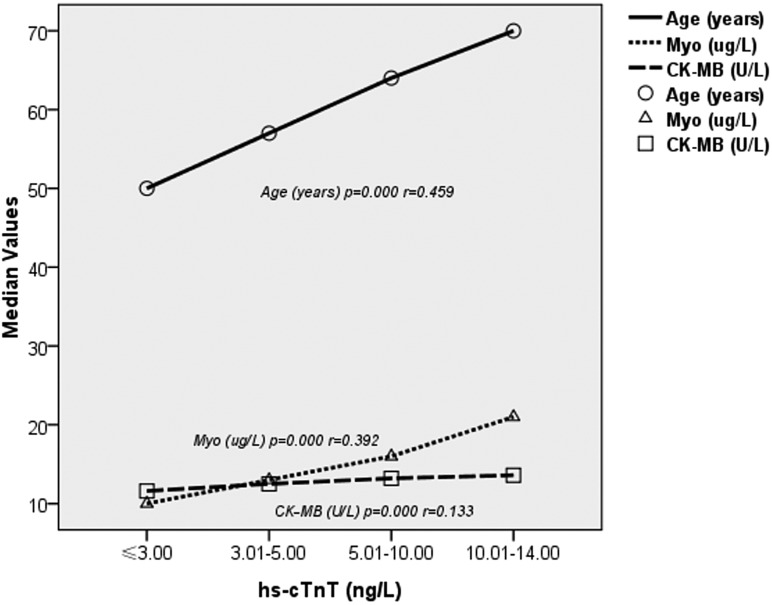
Nonparametric correlations between hs-cTnT and features of the study population

Table 3 and Figure 4 show the results of the Spearman analyses of the correlation of hs-cTnT with age, Myo and CK-MB. The results indicated that hs-cTnT was positively associated with age (*r* = 0.459, *p* = 0.000), Myo (*r* = 0.392, *p* = 0.000), and CK-MB (*r* = 0.133, *p* = 0.000).

**Table 3:** Nonparametric correlations between hs-cTnT and features of the study population

**Variables**	**Relationship coefficient**	***p* value**
Age (years)	0.459	0.000
Myo (ug/L)	0.392	0.000
CK-MB (U/L)	0.133	0.000

hs-cTnT, high-sensitivity cardiac troponin T; Myo, myoglobin; and CK-MB, creatine kinase-MB.

### Characteristics of the study population stratified by hs-cTnT values

**Figure 5:**
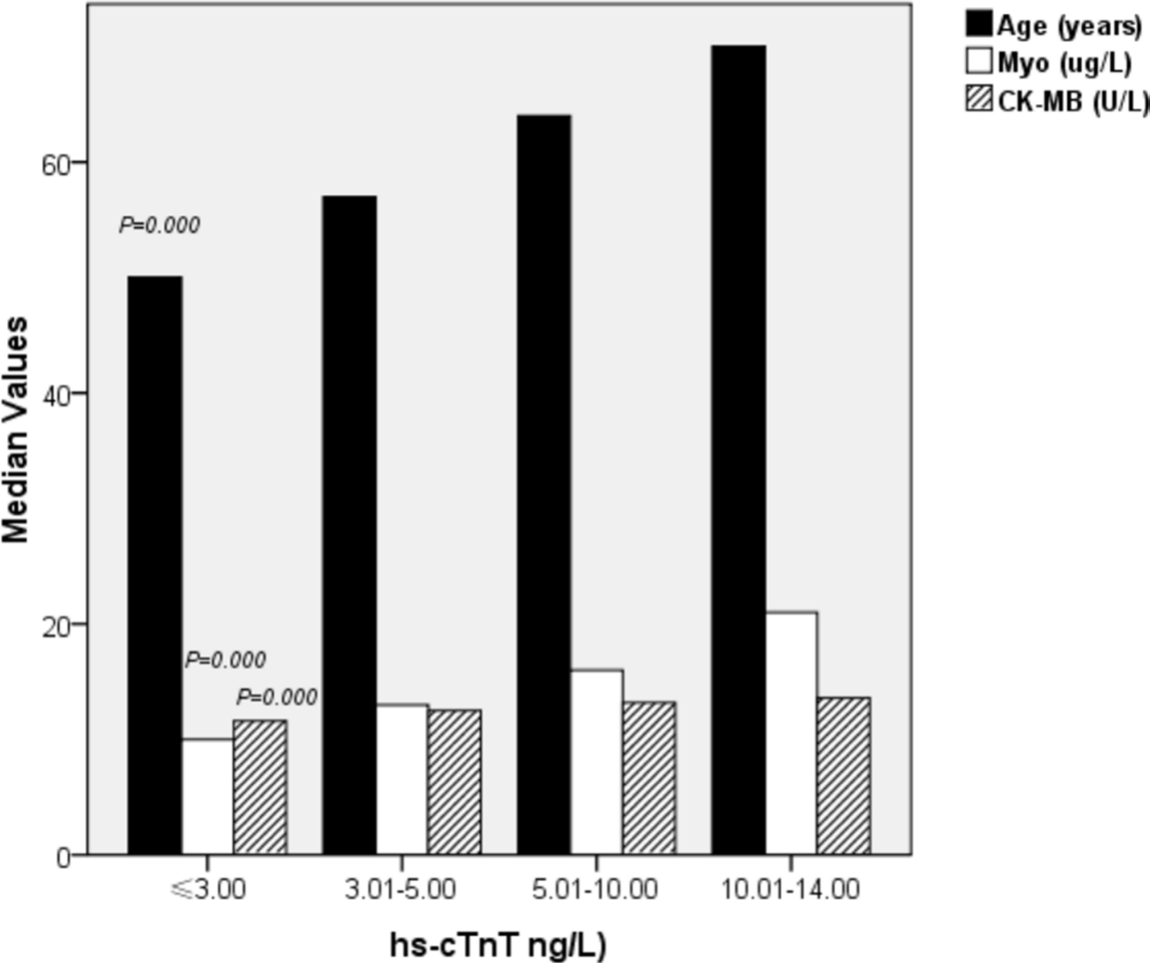
Characteristics of the study population stratified by values of hs-cTnT

Subjects were divided into 4 groups according to hs-cTnT values: ≤ 3.00 (LOB) (first group; *n* = 1316 subjects), 3.01–5.00 (LOD) (second group; *n* = 1152 subjects), 5.01–10.00 (third group; *n* = 2238 subjects) and 10.01–14.00 (fourth group; *n* = 879 subjects). The characteristics of the study population stratified by hs-cTnT values are shown in Table 4 and Figure 5. Myo (*p* = 0.000) and CK-MB (*p* = 0.000) concentrations significantly increased as hs-cTnT increased. Moreover, the group with higher hs-cTnT concentrations was older (*p* = 0.000).

**Table 4:** Characteristics of the study population stratified by values of hs-cTnT

Characteristics	hs-cTnT (ng/L)				Statistical parameter	*p* value
	–3.00 (*n =* 1316)	3.01–5.00 (*n =* 1152)	5.01–10.00 (*n =* 2238)	10.01–14.00 (*n =* 879)		
Gender (M/F) Age (years)	472/844 50.00 (37.00–60.00)	626/526 57.00 (45.00–65.00)	1311/927 64.00 (54.00–73.00)	571/803 70.00 (61.00–78.00)	234.249 1135.466	0.000 0.000
Myo (ug/L)	10.00 (8.00–15.00)	13.00 (9.00–18.00)	16.00 (11.00–24.00)	21.00 (14.00–36.00)	791.450	0.000
CK-MB (U/L)	11.60 (8.70–15.18)	12.50 (9.40–16.00)	13.20 (10.00–17.13)	13.60 (10.00–17.90)	89.199	0.000

hs-cTnT, high-sensitivity cardiac troponin T; Myo, myoglobin; and CK-MB, creatine kinase-MB.

## DISCUSSION

In a large cohort of 5585 consecutive subjects who presented with nontraumatic chest pain/discomfort to the inpatient, outpatient, or emergency department and who were not diagnosed with MI, we found that the hs-cTnT values below the 99th percentile were higher in men and increased with age in both men and women. In addition, the concentrations of the myocardial injury biomarkers, Myo and CK-MB were associated with hs-cTnT. To our knowledge, this study is the first to document that age and sex differences exist in patients with very low hs-cTnT, Myo and CK-MB concentrations.

In clinical practice, hs-cTnT was introduced as a highly sensitive and early biomarker of myocardial damage [2, 9, 10]. Concentrations of hs-cTnT below the 99th percentile (≤ 14 ng/L) have been suggested to have the potential to rule out MI at an earlier stage, and undetectable hs-cTnT (≤ 5 ng/L) has a higher NPV for MI [2, 6, 9–13]. Furthermore, patients who present to the ED with different hs-cTnT concentrations below the 99th percentile have completely different prognoses and clinical outcomes [8, 12, 14]. However, the clinical use of low hs-cTnT levels does not consider patient-related factors (namely, age and sex). To objectively and accurately assess patients with hs-cTnT concentrations below the 99th percentile, we explored the factors of sex and age in 5585 patients who underwent hs-cTnT assays who were included in our study.

In the present study, hs-cTnT values below the 99th percentile were positively associated with age (*r* = 0.459, *p* = 0.000) and increased with age among both men and women. These observations are in line with the results of recently published studies [15, 16] that showed higher hs-cTnT concentrations in the elderly. The vast majority of older patients who have ischemia disease also have various other complications; underlying diseases such as diabetes, high blood pressure and chronic kidney disease may cause an increase in hs-cTnT values, biasing the reference standard of low hs-cTnT concentrations for ruling out AMI and/or adverse prognostic implications. Our analysis suggests that the evaluation of low hs-cTnT concentrations in both research and clinic should take patients’ age into consideration.In a meta-analysis of three studies including 10723 patients, participants with hs-cTnT concentrations between the LOD and the LOB who were at higher risk were also older [8]. This results is entirely consistent with our finding that age was significantly different among patients with very low hs-cTnT concentrations. This result is also again consistent with the findings of a large retrospective analysis by Body et al., which suggested that use of the “rule out” strategy, in which the detection of very low hs-cTnT concentrations at admission seems to allow for rapid and safe exclusion of AMI, is restricted to patients < 65 years of age [13].

We have also presented data regarding sex differences in patients with low hs-cTnT concentrations. In our study, the massive dataest illustrated in Table 1 and Figure 2 shows that hs-cTnT values below the 99th percentile (14 ng/L) were higher in men (*p* = 0.000). Moreover, the proportion of males (*p* = 0.006) was greater than that of females in each quartile of age. In addition, males were younger (*p* = 0.000) and had higher Myo (*p* = 0.000) and CK-MB (*p* = 0.000). It was recently reported that use of a sex-specific 99th percentile cut-off hs-cTnT value (women, 9 ng/L; men, 15.5 ng/L) may increase its sensitivity for AMI diagnosis but may also cause an obligatory drop in specificity and an increased rate of false-positive hs-cTnT levels in women [17]. Our findings suggest that sex should still be considered an important patient-related factor influencing MI risk when using low hs-cTnT concentrations as a clinical diagnostic tool.

In a study conducted by Omland et al. [10], the authors noted that as hs-cTnT assays become more sensitive, low-level hs-cTnT elevations will be less specific for the determination of acute myocardial injury. Taking this consideration into account, we maintain that the association of hs-cTnT with other myocardial injury biomarkers, such as Myo and CK-MB, would increase the specificity and accuracy of MI detection. Use of the LOD of the high-sensitivity cardiac troponin I assay and plasma glucose to rule out AMI has a diagnostic sensitivity of 100% [18]. Therefore, our study provided information concerning the association of certain biomarkers. The data indicated that hs-cTnT was positively associated with Myo (*r* = 0.392, *p* = 0.000) and CK-MB (*r* = 0.133, *p* = 0.000). Additionally, the Myo (*p* = 0.000) and CK-MB (*p* = 0.000) concentrations increased as hs-cTnT increased. Furthermore, we found that age and sex differences also exist in patients with very low Myo and CK-MB concentrations. The adoption of decision limits for CK-MB based on gender (7.1 ng/mL for men and 5.4 ng/mL for women) to improve the specificity of MI detection has also been reported [19]. Thus, we suggest that a new multimarker strategy using low concentrations of hs-cTnT combined with other myocardial injury biomarkers could be effective for MI diagnosis.

In summary, the present study provides the first insights into age-sex differences in patients with hs-cTnT values below the 99th percentile upper reference limit worldwide. The strengths of this analysis include the large number of participants (5585 subjects, including 2980 males and 2605 females, aged 4–104 years) and the identification of interactions among sex, age, hs-cTnT, Myo and CK-MB. These findings have important implications for the ongoing development of hs-cTnT assays, illustrating that when evaluating the general population with any hs-cTnT concentration that is below the 99th percentile, age-sex differences should be taken into consideration. The results of the study also emphasize the necessity of improving the specificity and accuracy of the hs-cTnT assay in combination with assays for other myocardial injury biomarkers for acute myocardial injury in the general population.

## MATERIALS AND METHODS

### Study subjects

This study was conducted at the First Affiliated Hospital of Nanjing Medical University (Nanjing, China), a tertiary-care teaching hospital.

From January 1st, 2015 to October 31st, 2015, 5585 consecutive subjects (2980 males and 2605 females) aged 4–104 years who presented with non-traumatic chest pain/discomfort to the inpatient, outpatient, or emergency department and who underwent hs-cTnT, myoglobin (Myo) and creatine kinase-MB (CK-MB) testing at presentation, with hs-cTnT below the 99th percentile (≤ 14 ng/L), were eligible for enrollment. We excluded subjects with ST-segment elevation myocardial infarction (STEMI), since their diagnosis was not dependent on troponin levels. And, all participants were required to provide written informed consent. The experiments were performed in accordance with approved guidelines, and all experimental protocols were approved by the Ethics Committee of the First Affiliated Hospital of Nanjing Medical University. The study was conducted in accordance with the principles of the Declaration of Helsinki.

The diagnostic flow scheme which has been used for the diagnosis of AMI in the present study was as following [20]. AMI defines cardiomyocyte necrosis in a clinical setting consistent with acute myocardial ischaemia. A combination of criteria is required to meet the diagnosis of AMI, namely the detection of an increase and/or decrease of a cardiac biomarker, preferably high-sensitivity cardiac troponin, with at least one value above the 99th percentile of the upper reference limit and at least one of the following:

(1) Symptoms of ischaemia.
(2) New or presumed new significant ST-T wave changes or left bundle branch block on 12-lead ECG.
(3) Development of pathological Q waves on ECG.
(4) Imaging evidence of new or presumed new loss of viable myocardium or regional wall motion abnormality.
(5) Intracoronary thrombus detected on angiography or autopsy.

### Laboratory measurements

The biomarkers hs-cTnT, Myo and CK-MB are widely recognized as the most dynamic responders to myocardial injury [21]. Blood samples for hs-cTnT testing were collected in lithium heparin tubes or sodium heparin tubes stored at 2–8°C. Plasma was then immediately separated by centrifugation within 4 h of collection, and samples were frozen at −80°C. Measurement was performed using an hs-cTnT one-step electrochemiluminescence immunoassay on an Elecsys 2010 analyzer (Roche Diagnostics, Mannheim,Germany). The upper reference limit (URL) for this assay, defined as the 99th percentile cut-off value (14 ng/L), is from a study of 533 “apparently healthy” volunteers, and the lowest hs-cTnT concentration that can be measured with a coefficient of variation (CV) ≤ 10% using this assay is 13 ng/L [3]. The LOB defined as the highest apparent hs-cTnT concentration in an analyte-free sample tasted using this assay, was 3 ng/L. In addition, the LoD defined as the lowest actual concentration of hs-cTnT that can be reliably quantified in a given sample,was 5 ng/L [3, 22]. Samples for Myo testing were stored at 2–8°C and were assessed via an enzyme-linked fluorescence immunoassay on a VIDAS analyzer. The reference interval for Myo (95% of sample values), derived from a study of 197 patients without a personal history of heart disease, was 10–46 ug/L. At the same time, serum was separated by centrifugation within 4 h after the blood sample for CK-MB testing was collected. Samples were equilibrated to 18–28°C and immediately assessed using the VITROS chemistry products’ CK-MB slides on a VITROS 5600automatic biochemical analyzer [23].The detection interval for CK-MB was 2.7–300 U/L, and a single value for CK-MB was obtained by measurement at a short time point, and analyzed for clinical diagnosis.

### Statistical analysis

Data were analyzed using the Statistical Package for the Social Sciences (ver. 16.0; SPSS Incorporated, Chicago, IL, USA). Subjects were classified into 2 groups based on their gender and 4 groups according to the quartile of their age and their hs-cTnT values. Patients were classified into four groups according to the hs-cTnT concentrations using quartile values as the cut-off points; therefore, each group had a similar number of patients, minimizing any bias that may have been produced in the statistical analyses. Skewed data, including age, hs-cTnT, Myo, and CK-MB were expressed as medians and quartile ranges, and comparisons were performed using the Mann-Whitney U test and the Kruskal-Wallis H test. The categorical variable gender was compared among the patient groups using chi-squared analysis. The Spearman two-way test was used to assess the relationship of hs-cTnT with age, Myo, and CK-MB. All hypothesis testing was two tailed, and *p* < 0.05 was considered statistically significant.

## CONCLUSIONS

Age and sex differences were significant in patients with hs-cTnT values below the 99th percentile (< 14 ng/L), and the values were higher in men and the elderly. Moreover, interactions among sex, age, hs-cTnT, Myo and CK-MB were found. The clinical implications of our findings are that the use of very low hs-cTnT concentrations for ruling out AMI and/or adverse prognostic implications should take into account patients’ age and sex and should be combined with the analysis of other myocardial injury biomarkers. This approach could improve the specificity and accuracy of MI diagnosis, reducing the misclassification of patients and unnecessary hospital admissions.

### Limitations

There are a few limitations associated with this study. First, the study was performed at a single hospital, and therefore, it remains necessary to analyze findings at other centers, even though our results are similar to those of other studies [22, 14–17]. Second, we only analyzed the interactions among age, sex, low hs-cTnT concentrations, Myo and CK-MB. Presented baseline characteristics including medication usage, medical history are limited. Furthermore, there were no reliable data to derive an association between the various hs-cTnT concentrations below the 99th percentile and the clinical prognoses of the patients. Third, the ESC guidelines and Reichlin et al. have demonstrated that hs-cTnT measurement should always be used in conjunction with an assessment of patient history and ECG when excluding MI [2]. However, the present study did not discuss this combination. Lastly, although we included data from a large cohort of patients, these data are observational, so further interventional studies will be necessary.
